# *Crocus sativus* (saffron) in the treatment of female sexual dysfunction: a three-center, double-blind, randomized, and placebo-controlled clinical trial

**DOI:** 10.22038/AJP.2022.19714

**Published:** 2022

**Authors:** Ladan Kashani, Sahar Aslzadeh, Kamyar Shokraee, Ahmad Shamabadi, Borna Tadayon Najafabadi, Morteza Jafarinia, Sophia Esalatmanesh, Shahin Akhondzadeh

**Affiliations:** 1 *Infertility Ward, Arash Hospital, Tehran University of Medical Sciences, Tehran, Iran*; 2 *Psychiatric Research Center, Roozbeh Hospital, Tehran University of Medical Sciences, Tehran, Iran*; 3 *School of Medicine, Tehran University of Medical Sciences, Tehran, Iran*; 4 *Department of Health Research Methods, Evidence & Impact, McMaster University, Hamilton, Canada*; † * Equal first author*

**Keywords:** Crocus sativus, Randomized controlled trial Saffron, Sexual dysfunction

## Abstract

**Objective:**

One of the traditional aphrodisiacs used in various cultures is *Crocus sativus*, commonly called saffron. Previous studies have pointed to the possible applicability of saffron for sexual dysfunction in both men and women. This study investigates the effects of saffron capsules on female sexual dysfunction.

**Materials and Methods:**

This study was a parallel-group, double-blind, randomized, placebo-controlled clinical trial. Participants, who were married women between 18 and 55 years of age suffering from severe sexual dysfunction, were randomized to receive either 15 mg *Crocus sativus* capsules twice daily or placebo. The treatment continued for 6 weeks, and patients were evaluated every 2 weeks. The primary outcome was the change in the female sexual function index score. Other outcomes included the female sexual function index sub-domains.

**Results:**

Seventy

-four patients were equally randomized to each group, and 34 in each group completed the trial. Participants in both groups experienced improved total scores at each visit. However, a repeated-measures ANOVA revealed that time treatment differed between groups in favor of the saffron group (p=0.050). During the 6^th ^week follow-up, the saffron group had a 62% score improvement from baseline. Desire, lubrication, and satisfaction were female sexual function index domains in which saffron demonstrated superiority over placebo. The adverse event profile was similar for the groups, and no participant discontinued treatment.

**Conclusion:**

Findings of this study suggest that saffron might be a safe and effective option to ameliorate female sexual dysfunction. Further robust research is warranted.

## Introduction

Female sexual dysfunction (FSD) is regarded as a difficult-to-treat disorder despite its high prevalence, so that 43% of the female population has some form of sexual dysfunction (Shifren et al., 2008 ▶). Also, the prevalence in Iran seems to be in the same range (Tabaghdehi et al., 2017 ▶). In a study in 2006, FSD complaints were reported to be 64% for desire difficulties, 35% for orgasm difficulties, 31% for arousal difficulties, and 26% for sexual pain. The disorder based on the distress it causes to patients, is divided into mild, moderate, and severe (Hayes et al., 2006 ▶; Lodise, 2017 ▶). While the definition and the classification of this disorder have undergone substantial changes after the publication of the 5^th^ edition of the Diagnostic and Statistical Manual of Mental Disorders (DSM-5) (American Psychiatric Association, 2013 ▶), the treatment options are still limited to psychosocial interventions and a few pharmaceutical agents with inconsistencies in their applicability for this disorder (Lodise, 2017 ▶; Miller et al., 2018 ▶).

Unlike male sexual dysfunction, for which the etiology is relatively known, female sexuality is much less understood, as debilitating as it is (Basson et al., 2001 ▶; Brown et al., 2007 ▶; Goldstein, 2008 ▶). This unawareness might arise from the fact that FSD is multifactorial and includes biological, psychosexual, and contextual factors (Graziottin et al., 2009 ▶). The pathophysiological pathways leading to this disorder are yet to be identified. However, as with males, studies indicate that there are correlations between FSD and cardiovascular diseases (Jackson, 1999 ▶; Kostis et al., 2005 ▶; Rosman et al., 2014 ▶). The pathophysiology of sexual dysfunction is similar to that of cardiovascular diseases, involving endothelial dysfunction, subclinical inflammation, atherosclerosis, and vascular damages. On the other hand, the mental factors that arise following cardiovascular events affect patients’ sex lives (Imprialos et al., 2018 ▶). Also, other pieces of evidence point to a relationship between FSD and an imbalance in the neurotransmitter system, which further suggests that using neuro-effective drugs might help ameliorate sexual problems in women (Hensley and Nurnberg, 2002 ▶).

One of the traditional aphrodisiacs used in various cultures is saffron (*Crocus sativus*). Previous studies have pointed to the possible applicability of saffron for sexual dysfunction in both men and women (Hosseinzadeh et al., 2008 ▶; Kashani et al., 2013 ▶; Shamsa et al., 2009 ▶). Furthermore, other studies have indicated that saffron can be useful, at least as a supplement, in treating depressive and anxiety disorders (Agha-Hosseini et al., 2008 ▶; Akhondzadeh Basti et al., 2007 ▶; Akhondzadeh et al., 2004 ▶; Akhondzadeh et al., 2005 ▶). Saffron has been seen to possess antinociceptive effects, which might reduce pain-related discomfort during a sexual relationship through peripheral blockage of pain detection (Hosseinzadeh and Younesi, 2002 ▶). Putting together the favorable safety profile and central nervous system actions of saffron, *C. sativus* seems promising as a potential supplement in treating FSD.

This study hypothesized that the administration of saffron supplements improves sexual function among women suffering from severe FSD. Therefore, we aimed to answer the question of whether if, in women with severe FSD, the administration of saffron capsules would improve FSD compared with placebo.

## Materials and Methods

**Study design and setting**

The current study was a parallel-group, double-blind, randomized, placebo-controlled trial. Participants were randomly allocated to receive either saffron or placebo for 6 weeks. The participants were recruited from three centers in Tehran, Iran, including Imam Khomeini Hospital Psychosomatic Outpatient Clinic, Shah Abadi Health Center, and Emami Health Center (all affiliated with Tehran University of Medical Sciences), from August 2018 to January 2021. The institutional board of Tehran University of Medical Sciences reviewed and approved the trial protocol before the study (IR.TUMS.VCR.REC.1397.223). The registered protocol can also be found in the Iranian Registry of Clinical Trials (IRCT20090117001556N110). This trial was conducted in accordance with the Helsinki declaration and its subsequent revisions. All participants provided informed consent before enrollment. They were explicitly informed about the trial design and their rights, such as their choice to withdraw at will without affecting their healthcare provisions.

**Participants**

Consecutive married women between the ages of 18 and 55 with a complaint of sexual dysfunction who were referred to the clinic in our study were screened using a convenient sampling method. To establish the presence of severe sexual dysfunction, patients completed the Female Sexual Function Index (FSFI) questionnaire. Patients with a total score of 16 or under who were identified as sexually active, were eligible (Rosen et al., 2000). Our study excluded patients if any of the following existed: (a) uncontrolled psychiatric disorder based on the Diagnostic and Statistical Manual of Mental Disorders, 5th Edition (DSM-5), (b) hormonal or genetic disorders with effects on female sexual function, (c) substance abuse, (d) pregnancy, or (e) lactation.

Two research assistants, affiliated with the study and located in the clinics, initially filled the FSFI for each potential participant. Afterward, an expert psychiatrist systematically interviewed those meeting the inclusion criteria to check for the exclusion criteria. Also, an expert psychiatrist with extensive experience with the FSFI questionnaire trained the research assistants on using the FSFI questionnaire. Furthermore, the research assistants gathered and stored participants data securely. A gynecologist was also involved in all referral clinics.

**Intervention**

Participants were randomly allocated to the study groups. Those in the saffron group received 15 mg of saffron capsules twice daily. The saffron extract preparation method is detailed elsewhere (Akhondzadeh et al., 2010 ▶). In the placebo group, participants received starch-based placebo capsules, identical to the saffron capsules, twice daily. Also, there was no difference in taste and smell between the capsules. Patients in both groups received instructions to take their capsules after a meal with a glass of water. The instructions also encouraged them to avoid other treatments aimed to improve sexual function during the trial. The research assistants provided uniform instructions to the participants, both written and verbally. Treatment continued for 6 weeks, and participants were followed at weeks 2, 4, and 6. Keeping clinic appointments, phone calls, and pill counts were performed to evaluate participants’ adherence. During each follow-up, patients received reminders about the importance of adhering to the treatment and instructions mentioned above.

**Outcome**

The primary outcome of interest in our study was an improvement in the FSFI questionnaire total score during the trial as a reliable and validated indicator of female sexual function. Therefore, we focused on the between-group differences in changes of the FSFI score from baseline. This questionnaire is a valid and reliable 19-item tool that evaluates various domains of female sexual function. These domains include desire, arousal, lubrication, orgasm, satisfaction, and pain (Rosen et al., 2000 ▶). Each domain can score up to 6 points, while 1.2 and 0.8 are the only non-zero minimums (for desire and satisfaction, respectively). Therefore, the total score ranges from 2 to 36, with higher scores indicating better function. This sensitive patient-reported outcome measure was originally designed for use in FSD trials (Rosen, 2002 ▶). We used a validated Persian translation of the English questionnaire (Fakhri et al., 2012 ▶). We also reported each domain score separately.

We also recorded the adverse events experienced by the participants during the trial as secondary outcomes. We developed a 25-item checklist of possible adverse events to document these events systematically. Open questions about any other adverse events or concerns completed the checklist. After the initial recording of the baseline variables, the study’s research assistants contacted participants every 2 weeks by phone. Participants completed the FSFI questionnaire and the adverse events checklist in each contact. They also received reminders about adhering to the treatment and the study protocol. The research assistant, in charge of data gathering, obtained training from an expert psychiatrist on the study protocol and working with the FSFI questionnaire.

**Randomization and blinding**

A party not involved elsewhere in the trial generated a random sequence electronically. Based on that random sequence, saffron and placebo capsules were placed in consecutively numbered packages. All packages were identical other than their number. Each package contained treatment capsules and an information sheet on the trial design, instructions on using the capsules, and a list of other interventions to avoid during the study. The saffron and placebo capsules were, as well, identical in shape, size, color, taste, and smell. Participants consecutively received treatment packages. In this manner, participants, physicians, outcome assessors, and package dispensary staff were blinded. The allocation list was not revealed to any engaged party until after the last participant completed the trial. Statistical analysis was unblinded according to a plan set *a priori*.

**Sample size and statistical analysis**

Based on the results of a previous study, we assumed a standard deviation (SD) of 4 for the FSFI total score (Kashani et al., 2013 ▶). Since a minimal clinically significant difference for the FSFI score has not been calculated yet, we assumed a between-group difference of 3 units for the outcome. Considering a type I error of 5%, a power of 80%, and a 1:1 allocation ratio yielded a sample size of 58. We used a conservative estimate of a 20% dropout rate to preserve power. The final sample size came to 74.

The statistical analysis plan was set *a priori*. To describe our variables, we used meanSD and mean difference [95% CI] for continuous variables and frequency (percentage) for categorical variables. Chi-square tests or Fisher’s exact test, when appropriate, compared the categorical outcomes between groups. Furthermore, to compare each follow-up FSFI score with its baseline within each group, we conducted paired-sample t-tests. Also, repeated-measures analysis of variance (ANOVA) was used to compare changes of FSFI scores between groups. This analysis reveals if the treatment time interaction is significantly different between groups. Greenhouse-Geisser correction for the degree of freedom was used whenever the sphericity of data could not be assumed. A p-value level equal to or less than 5% indicated significance.

## Results

**Participants**

**Figure 1:**
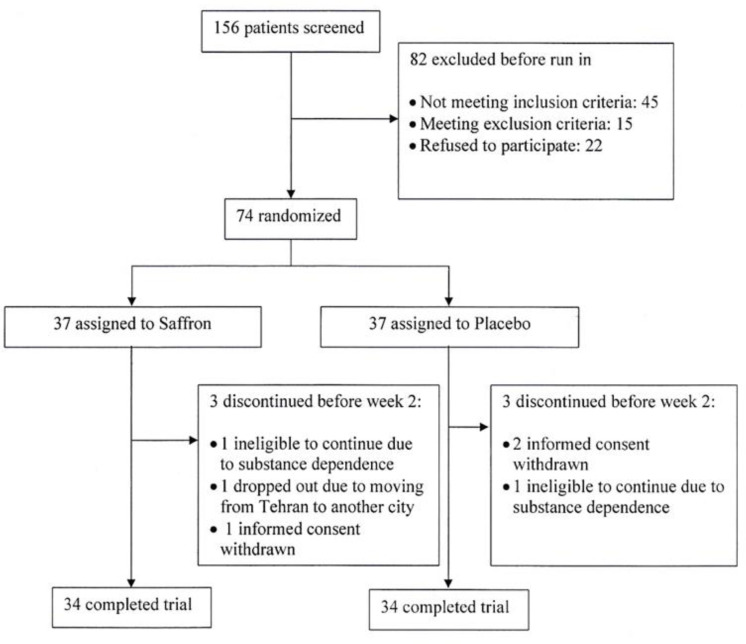
Trial flow diagram

One hundred fifty-six patients were screened for the eligibility criteria, and 74 were equally randomized to saffron and placebo. In each group, 3 participants left the study before the first follow-up in the second week. Thirty-four participants in each group completed the trial. Figure 1 presents the study flow diagram and reasons for dropouts. Fortunately, there are no missing outcome data for participants who completed the trial. Our participants had a mean age of 36.68.0 years ranging from 18 to 53 years of age. They also had an overall baseline FSFI total score mean of 13.92.5. There was no difference in the demographic characteristics between the two groups. Table 1 details the baseline characteristics for each treatment group.

**Table 1:** Baseline characteristics of the patients

Variable		Treatment Group							
		Saffron (n=34)				Placebo (n=34)			
		Mean	SD	Count	%	Mean	SD	Count	%
Age, years		37	9			37	7		
Marital age, years		15.2	9.3			15.3	8.9		
Education	Below high-school			13	38.2			19	55.9
	high-school			13	38.2			10	29.4
	academic			8	23.5			5	14.7
FSFI domain	total	14.04	2.7			13.76	2.34		
	desire	1.97	0.85			2.08	0.62		
	arousal	1.81	0.66			1.81	0.70		
	lubrication	2.54	1.17			2.40	0.83		
	orgasm	2.00	0.86			2.11	0.63		
	satisfaction	2.05	0.89			2.23	0.92		
	pain	3.32	1.75			3.15	1.24		

SD: Standard deviation, FSFI: Female sexual function index

**FSFI Score**

**Figure 2:**
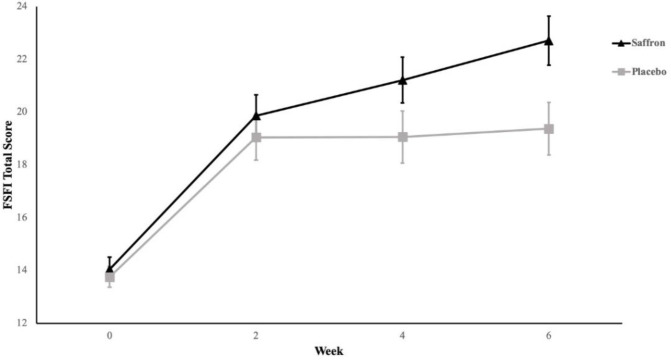
Saffron and placebo total FSFI scores during the trial. Error bars represent the standard error of the means

Both groups experienced significantly better total FSFI scores from the first follow-up. However, the mean score for the saffron group improved in subsequent follow-ups as well. Table 2 demonstrates score changes from baseline value in each group. After 6 weeks, the saffron group participants experienced 8.7 points (95% CI [6.6, 10.7]) of improvement from baseline. It corresponds to a 62% improvement from the initial value. To establish if the score change was different between groups, we carried out repeated-measure ANOVA. This analysis revealed that time treatment was different between groups in favor of the saffron group (Greenhouse-Geisser F (2.0, 47.7) = 3.1, p =0.050). Note that this significance level is borderline. Figure 2 depicts the changes in FSFI total scores in each group over the course of the study.

Regarding individual domains of the FSFI questionnaire, as Table 2 indicates, changes from baseline in each intervention group were statistically significant. The greatest improvement (absolute difference) experienced for the saffron group participants was in the satisfaction domain. On the other hand, the least yet meaningful change was for the pain domain. Similar to the total score analysis, we used repeated-measure ANOVA to investigate the between-group difference in domain score changes from baseline. Details of the analysis for each domain are incorporated in Table 3. Domains in which the saffron group experienced significantly higher improvement than placebo include desire, lubrication, and satisfaction.

**Adverse events**

The most frequently observed adverse events (approximately 10% of the participants) in this trial were headaches and nausea. Table 4 details the adverse events reported in each group. The frequency of adverse events was similar between the groups. Importantly, the profile of these events was clinically acceptable, and none of the participants deemed any of the adverse events intolerable. All participants continued their assigned treatment until the end of the trial.

**Table 2:** IIEF scale scores compared to the baseline values

		Treatment Group							
		Saffron (n=34)				Placebo (n=34)			
FSFI domain	week	MD*	SEMD	95% CI		MD*	SEMD	95% CI	
				LL	UL			LL	UL
Total Score	2 weeks	5.81	0.90	3.98	7.65	5.28	0.85	3.55	7.02
	4 weeks	7.17	0.90	5.34	9.00	5.30	0.94	3.39	7.21
	6 weeks	8.66	1.00	6.62	10.71	5.61	1.03	3.51	7.71
Desire	2 weeks	0.99	0.16	0.66	1.31	0.49	0.14	0.20	0.77
	4 weeks	1.31	0.18	0.96	1.67	0.61	0.17	0.27	0.95
	6 weeks	1.36	0.22	0.92	1.81	0.67	0.15	0.37	0.97
Arousal	2 weeks	1.07	0.22	0.63	1.51	0.76	0.17	0.41	1.10
	4 weeks	1.29	0.21	0.86	1.73	0.88	0.16	0.56	1.21
	6 weeks	1.47	0.22	1.03	1.91	1.06	0.21	0.64	1.49
Lubrication	2 weeks	0.83	0.21	0.39	1.26	0.70	0.18	0.33	1.07
	4 weeks	1.34	0.24	0.86	1.82	0.75	0.19	0.37	1.14
	6 weeks	1.65	0.25	1.15	2.15	0.89	0.19	0.50	1.27
Orgasmic Function	2 weeks	1.22	0.24	0.73	1.72	1.05	0.18	0.68	1.41
	4 weeks	1.45	0.25	0.94	1.97	1.03	0.22	0.58	1.48
	6 weeks	1.74	0.24	1.26	2.23	1.14	0.22	0.69	1.60
Satisfaction	2 weeks	1.21	0.24	0.73	1.70	0.97	0.20	0.56	1.38
	4 weeks	1.58	0.23	1.10	2.05	0.96	0.21	0.53	1.40
	6 weeks	1.94	0.24	1.45	2.42	0.98	0.21	0.56	1.41
Pain	2 weeks	0.92	0.20	0.51	1.32	1.03	0.26	0.50	1.56
	4 weeks	1.16	0.20	0.76	1.56	0.82	0.26	0.30	1.34
	6 weeks	1.35	0.22	0.90	1.81	0.88	0.28	0.32	1.44

FSFI, Female Sexual Function Index; MD, mean difference; SEMD, standard error of mean difference; CI, confidence interval; LL, lower limit; UL, upper limit.

*MD postbaseline value baseline value

**Table 3:** Repeated-measure ANOVA for FSDI score and comparison between the groups

FSFI Domain	GG Correction	df	Mean Square	F	p-value
Desire	Yes	2.313	2.388	4.755	0.007
Arousal	Yes	2.146	0.893	1.324	0.270
Lubrication	Yes	2.118	3.208	4.044	0.018
Orgasm	Yes	1.976	1.817	2.016	0.138
Satisfaction	Yes	2.113	4.216	5.046	0.007
Pain	Yes	2.215	1.762	1.811	0.163

FSFI, Female Sexual Function Index; GG, Greenhouse-Geisser; df, degree of freedom.

**Table 4:** Frequency of adverse events

	**Treatment group**				
	Saffron (n=34)		Placebo (n=34)		
	Count	%	Count	%	p-value
**Headache**	4	11.8	4	11.8	1.000
**Insomnia**	2	5.9	2	5.9	1.000
**Dry mouth**	1	2.9	1	2.9	1.000
**Nausea**	3	8.8	2	5.9	1.000
**Constipation**	1	2.9	2	5.9	1.000
**Sweating**	2	5.9	2	5.9	1.000
**Vomiting**	1	2.9	1	2.9	1.000

## Discussion

This study demonstrated the superiority of saffron over placebo in improving the overall female sexual function score. However, since our results showed borderline significance, their interpretation requires caution. Participants also experienced a meaningful improvement regarding pain, lubrication, and satisfaction domains of the FSD scale used. Taking note of the similarity between the groups’ baseline values and study design, we can attribute the observed difference to the administration of saffron. Furthermore, reported adverse events demonstrated a safe 

and acceptable profile among the participants. The importance of this tolerability reveals itself, considering the long-term and challenging nature of this disorder.

Unfortunately, the literature on saffron for FSD is poor. In a 2019 systematic review, Ranjbar and Ashrafizaveh summarized the studies addressing saffron’s effects on both male and female sexual dysfunction (Ranjbar and Ashrafizaveh, 2019 ▶). They only found two randomized trials that included women with FSD. One study was a trial previously conducted by the current study’s research team that included female patients with selective serotonin reuptake inhibitor (SSRI)-induced sexual dysfunction (Kashani et al., 2013 ▶). The other study, however, included both men and women with coronary artery disease and evaluated the effects of saffron and its constituents on sexual desire improvement (Abedimanesh et al., 2017 ▶). The heterogeneous nature of the studies makes it difficult to interpret their results simultaneously. 

The current study results were also observed in our previous randomized, placebo-controlled trial assessing the effects of saffron for the treatment of fluoxetine-induced sexual dysfunction. The study assessed the sexual function improvement of 38 women who were suffering from fluoxetine-induced sexual dysfunction after the administration of saffron compared to placebo over 4 weeks. Similar to the current study, the total FSFI had improved significantly superior to placebo; however, findings of that study regarding each domain of the questionnaire are not completely consistent with the present results (Kashani et al., 2013 ▶).

On the other hand, the other trial included in the mentioned review could not find a significant improvement in sexual desire among those receiving saffron or its constituents compared with placebo. This trial randomized 30 men and 28 women, all with coronary artery disease and no specific restriction on baseline sexual desire, into three arms consisting of saffron extract, saffron constituents, and placebo. Participants received treatment for 8 weeks and were evaluated for improvement in sexual desire. The study reported findings aggregately for men and women. The results indicated that although improvement in desire score on the Hulbert Index of Sexual Desire (HISD) was observed, the study was unable to detect statistical significance between treatment groups and placebo (Abedimanesh et al., 2017 ▶).

To better understand why saffron might improve FSD, we would like to note the effects of depression on sexual function. Depression detrimentally affected sexual function in women in several other studies (Frohlich and Meston, 2002 ▶; Waldinger, 2015 ▶). In a 2018 review, Basson and Gilks argued that the incidence of sexual dysfunction is higher in depressed women, with depressed women being 3.12 more likely to suffer from FSD (Basson and Gilks, 2018 ▶; Mitchell et al., 2013 ▶). The dysfunctions caused by depression were more prominent in the pain, lubrication, and arousal domains of FSFI as studied by Mahmoud et al. (Mahmoud et al., 2018). It, thus, can be hypothesized that the established anti-depressive properties of saffron might ameliorate sexual dysfunction (Akhondzadeh Basti et al., 2007 ▶; Akhondzadeh et al., 2004 ▶; Akhondzadeh et al., 2020 ▶; Akhondzadeh et al., 2005 ▶; Milajerdi et al., 2018 ▶). A similar rationale can also be laid out for the efficacy of saffron as an anxiolytic agent and its relation to improving sexual dysfunction (Lo and Kok, 2018 ▶).

Moreover, amelioration of pain-related problems can be, in part, explained by the effects of saffron on the opioid system. Studies have linked intercourse-related pain to the activity of opioids and neuropeptides (Wilson et al., 2009 ▶). Saffron, interestingly, possesses antinociceptive properties that can be reversed by naloxone, suggesting opioid mediatory effects (Hosseinzadeh and Younesi, 2002 ▶). Furthermore, Hosseinzadeh and Jahanian found saffron to be efficacious in opioid withdrawal syndrome, in part accounting for improvement in the pain domain of FSFI seen in this study (Hosseinzadeh and Jahanian, 2010 ▶).

Along with its strengths, this study has limitations. Since we used a continuous measure, sample size calculations yielded a low sample size. The recruited number of participants is considered a small sample size. This issue brings about challenges and pitfalls, such as limited power for ensuring the absence of rare adverse events. Also, as noted earlier, the statistical analysis used to compare saffron and placebo just yielded borderline significance. Although we had set our analysis plan a priori, the reproducibility of similar results should be tested in further trials. However, this borderline difference can be due to the small sample size.

From an interpretation point of view, scales that are less commonly used by general clinicians can be challenging to understand. One suggested method to overcome this obstacle is to use minimal clinically significant difference, which identifies what changes in a scale are clinically meaningful. Nonetheless, this value is not currently available in the literature. Furthermore, the sensitivity of the FSFI questionnaire for improvements after treatment has not been thoroughly investigated.

Finally, FSD, its etiology, and its risk factors are far from well-known. Therefore, numerous possible confounders may impose some bias on the relation being considered in this study. We tried to tackle this issue by using a randomized design and measuring as many baseline factors as clinically justified and feasible to ensure enough randomization. Albeit, other baseline variables that can potentially confound the results might remain unaddressed.

The results of our study demonstrated an improvement, superior to placebo, in the overall indicator of female sexual function among those who received saffron. The superiority of this improvement was borderline significant over placebo. Also, the sub-domains of female sexual function with meaning improvement included desire, lubrication, and satisfaction. Importantly, we did not observe any intolerable adverse events in any participants. Therefore, we suggest that saffron might improve sexual dysfunction among women with FSD. Based on our results and other studies, we also believe that saffron is a safe and acceptable medication.

## Conflicts of interest

The authors have declared that there is no conflict of interest.
